# Psychopathological Predictors of Indirect Self-Destructiveness in Patients with Schizophrenia

**DOI:** 10.1007/s11126-015-9370-6

**Published:** 2015-05-17

**Authors:** Konstantinos Tsirigotis, Wojciech Gruszczyński, Marta Tsirigotis-Maniecka

**Affiliations:** Department of Psychology, Jan Kochanowski University in Kielce, Piotrków Trybunalski Branch, Słowackiego 114/118, 97-300 Piotrków Trybunalski, Poland; Institute of Applied Psychology, Social University of Sciences, Lodz, Poland; Division of Organic and Pharmaceutical Technology, Wroclaw University of Technology, Wrocław, Poland

**Keywords:** Indirect self-destructiveness, Schizophrenia, Psychopathological traits/symptoms, Prediction

## Abstract

Behaviours causing harm to the individual are generally called self-destructive behaviours. For some time now, direct/acute self-destructiveness has been distinguished from indirect/chronic self-destructiveness. Indirectly self-destructive behaviours occur not only in healthy people (examined in most of the studies) but also in mentally ill individuals, which has not been researched. The aim of this study has been to explore psychopathological (clinical) predictors of indirect self-destructiveness in patients with schizophrenia. Research was conducted among 200 patients suffering from paranoid schizophrenia (Sc) (according to ICD-10); average age: 37.15 (27–58) years. To assess indirect self-destructiveness, the Polish version of the “Chronic Self-Destructiveness Scale” was applied, whereas, to examine psychopathological characteristics, the Polish version of the “Minnesota Multiphasic Personality Inventory-2” was used. The correlation-regression procedure was followed. There were many statistically significant correlations, among which the strongest association occurred between indirect self-destructiveness and Sc and paranoia (Pa) scales (0.522 and 0.435 respectively). Significant predictors were found to be schizophrenia (Sc; R: 0.545; β: 0.412), lack of ego mastery, conative (Sc2b; R: 0.633; β: 0.632), and persecutory ideas (Pa1; R: 0.506; β: 0.335). schizophrenic disorders were a predictor explaining the indirect self-destructiveness syndrome in the patients. That aspect of psycho(patho)logical functioning, i.e. indirect self-destructiveness, which is strongly associated with schizophrenic and paranoid symptoms/disorders, should be considered in therapeutic work as well.

## Introduction

Behaviours causing harm to the individual are generally called self-destructive behaviours. Until recently, the term “self-destructiveness” was primarily (if not exclusively) understood as direct self-destructiveness, whose manifestations are self-mutilations or suicides. For some time, however, another type of self-harm has been distinguished, i.e. indirect or chronic self-destructiveness [1–3].

Although the issue of directly self-destructive behaviours (i.e. suicides, self-injuries etc.) is clear and does not arouse doubts, the less acute and “subtle” forms of harming oneself or lowering the quality of one’s life or shortening it, are not immediately perceptible (risky behaviours, substance abuse, addictions, negligence etc.). In general, less attention is given to them, particularly, since many of them are considered commonly (or at least often) occurring behaviours and, therefore, normal ones. Research into the area of indirect (chronic) self-destructiveness began in the 1980s and concerned mainly (if not solely) mentally healthy people.

Chronic self-destructiveness is defined as behaviour involving a generalized tendency to engage in acts that increase the probability of experiencing negative future consequences and/or reduce the probability of attaining positive future ones [1]. Indirect (chronic) self-destructiveness is also considered behaviour whose likely negative consequences are mediated by additional factors, while the relationship between behaviour and harm is considered probable. Indirect (chronic) self-destructiveness understood in that way includes not only undertaking but also abandoning (commission or omission of) acts; that is related to engaging in dangerous and aggravated risk situations, or neglecting one’s health and safety. Whereas acute/direct self-destructive behaviour involves conscious and wilful intent to self-inflict painful and injurious acts, sometime with fatal consequences, chronic/indirect self-destructiveness refers to actions extended over a period of time and situations, with the individual being unaware of or disregarding their long-term harmful effects [2, 3].

Indirectly self-destructive behaviours occur not only among healthy individuals, but also among mentally ill ones [4]. In the case of the mentally ill, indirectly self-destructive behaviours may involve (alternate with) psychotic symptoms or syndromes.

The aim of this work has been to explore psychopathological (clinical) predictors of indirect self-destructiveness in individuals with schizophrenia.

## Methods

The permission of the Bioethics Committee of the Medical University of Lodz was obtained before starting the research. The Helsinki Declaration recommendations were followed. The survey was anonymous and participation was voluntary. Before the survey, patients’ consent was obtained.

## Participants

In order to achieve the objective of the study, a group of 200 patients (83 females, 117 males) with paranoid Sc (according to ICD-10 criteria) was examined, aged 27–58 (mean age: 37.15) years. The patients were clinically stable (in remission), had not been hospitalized in the previous 12 months, and had been taking the same medication for at least 6 months. None of the patients was in relapse; the patients were in at least partial remission, which facilitated conducting the research. The characteristics of the study group are presented in Table 1.

**Table 1:** Characteristics of sample study

Variable	n	%
Sex		
Female	83	41.50
Male	117	58.50
Age		
Mean ± SD	37.15 ± 5.10	
Range	27–58	
Educational level		
Elementary	35	17.50
Trade	53	26.50
Secondary	92	46.00
University	20	10.00
Marital status		
Married	81	40.50
Divorced	15	7.50
Single	91	45.50
Widow/er	13	6.50
Residency		
Urban	110	55
Rural	29	45

## Materials

The Polish version of the Minnesota Multiphasic Personality Inventory-2 (MMPI-2) was used for assessing the intensity of psychopathological symptoms. In one survey, it is possible to acquire information on the most important personality dimensions as well as psychopathological symptoms; besides, it assesses the degree of similarity of the examined person’s traits to those characteristic of a given disease. Initially, the tool examined traits categorized into four validity scales and nine clinical scales. The clinical scales were: (1) hypochondriasis (Hd); (2) depression (D); (3) hysteria (Hy); (4) psychopathic deviate (PD); (5) masculinity–femininity (MF); (6) paranoia (Pa)—introduced to rate the clinical paranoid syndrome and leading to diagnoses of Sc or paranoid condition; (7) psychasthenia (Pt); 8) Scassessing the similarity of the subject to the patient with schizophrenia; (9) hypomania (Ma); 0) social introversion (SI). In the evolution of the MMPI, additional indices and indicators were developed and defined as supplementary scales and subscales of the clinical scales.

Among the subscales of the clinical scales by Harris and Lingoes, components of Pa and Sc are of the significance, i.e.: Pa1: persecutory ideas; Pa2: poignancy; Pa3: Naiveté; Sc1A: social alienation; Sc1B: emotional alienation; Sc2A: lack of ego mastery, cognitive; Sc2B: lack of ego mastery, conative; Sc2C: lack of ego mastery, defective inhibition; Sc3: Bizarre sensory experiences [5].

In order to assess indirect (chronic) self-destructiveness, the Polish version of the “Chronic Self-Destructiveness Scale” (CS-DS) by Kelley in Suchańska’s adaptation was used.

For the purpose of examining chronic self-destructiveness as a generalized tendency, Kelley constructed a research tool comprising four groups or categories of behaviours: carelessness, poor health maintenance, evidence of transgression, and lack of planfulness. The ultimate version was made up of an internally consistent set of 52 items with the total obtained score informing about the intensity of indirect self-destructiveness [1]. The Polish version of the scale and the original one are characterized by high reliability (Cronbach’s alpha, α: 0.811) and validity (0.823) [2, 3].

In order to assess relationships (associations) between psychotic symptoms and syndromes and indirect self-destructiveness, the correlation-regression procedure was applied.

Obtained quantitative data were subjected to a statistical analysis by means of the *Statistica PL 10.0 for Windows* [6] statistical package. The data were analysed using the mean, standard deviation, correlation coefficient (Kendall Tau, *τ*) and stepwise multiple regression analysis; p ≤ 0.05 was considered statistically significant.

## Results

The study results indicated that the intensity of indirect self-destructiveness in patients with Sc was within the average range (mean: 125.345; standard deviation: 21.521).

**Fig. 1:**
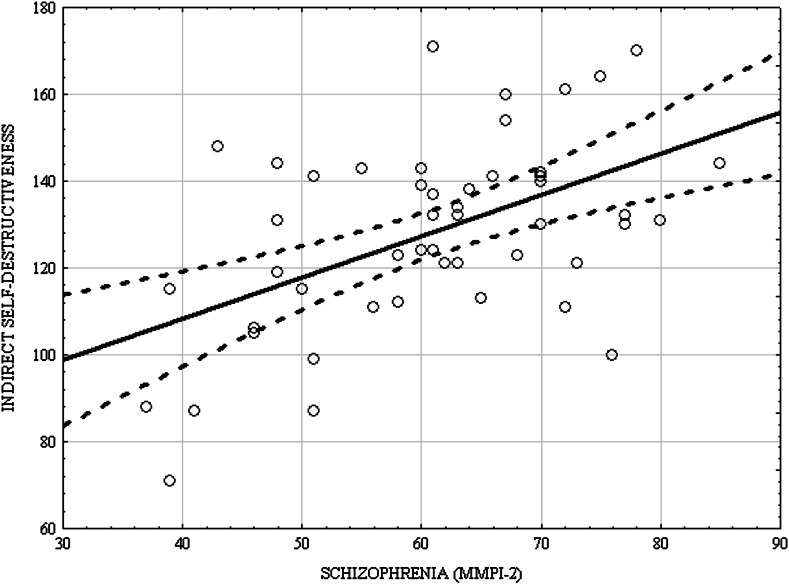
Scatterplot matrix of scores in the CS-DS and Sc (MMPI-2)

**Fig. 2:**
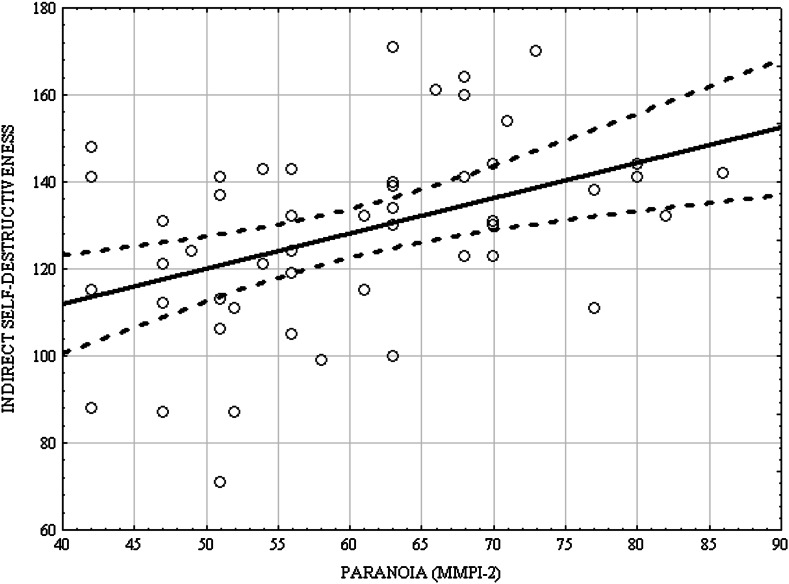
Scatterplot matrix of scores in the CS-DS and Pa (MMPI-2)

Table 2 and Figs. 1 and 2 show correlation coefficients between the patients’ scores on the MMPI-2 and CS-DS scales. As can be seen, there were many statistically significant correlations, out of which the strongest relationship occurred between indirect self-destructiveness and the scales of Sc and Pa (0.522 and 0.435 respectively). Apart from that, indirect self-destructiveness statistically significantly correlated with all the subscales of the Sc and Pa clinical scales; it was only for the Pa3 (Naiveté) subscale that the coefficient had the minus sign (−0.415).

**Table 2:** Correlations between patients’ with schizophrenia scores in MMPI-2 and CS-DS scales

MMPI-2 clinical scales and subscales	Indirect self-destructiveness (CS-DS)
Hd (Hypochondriasis)	0.215
	ns
D (Depression)	0.045
	ns
Hy (Hysteria)	0.005
	ns
PD (Psychopathic deviate)	0.340
	p: 0.01
MF (Masculinity–femininity)	0.043
	ns
Pa (Paranoia)	0.435
	0.002
Pt (Psychasthenia)	0.343
	p: 0.01
Sc (Schizophrenia)	0.522
	p: 0.0001
Ma (Hypomania)	0.351
	p: 0.01
SI (Social introversion)	0.054
	ns.
Pa1 (Persecutory ideas)	0.463
	0.004
Pa2 (poignancy)	0.441
	p: 0.005
Pa3 (Naiveté)	−0.415
	p: 0.01
Sc1A (Social alienation)	0.412
	p: 0.01
Sc1B (Emotional alienation)	0.485
	p: 0.002
Sc2A (Lack of ego mastery, cognitive)	0.471
	p: 0.003
Sc2B (Lack of ego mastery, conative)	0.635
	p: 0.00001
Sc2C (Lack of ego mastery, defective inhibition)	0.389
	p: 0.01
Sc3 (Bizarre sensory experiences)	0.467
	p: 0.003

Table 3 presents results of the stepwise multiple regression analysis, in which the dependent variable was indirect self-destructiveness and independent variables were the clinical scales and subscales of MMPI-2. All the clinical scales and subscales were included in the initial regression equation. As shown by the table, not all variables remained in the regression equation and, among those that remained, not all turned out to be significant for indirect self-destructiveness. Significant predictors were found to be schizophrenia (Sc; R: 0.545; β: 0.412), lack of ego mastery, conative (Sc2b; R: 0.633; β: 0.632) and persecutory ideas (Pa1; R: 0.506; β: 0.335).

**Table 3:** Stepwise multiple regression (Clinical scales/subscales of MMPI-2 and indirect self-destructiveness, CS-DS)

Dependent (criterion) variable: indirect self-destructiveness (CS-DS)						
Coefficient of multiple regression R = 0.545						
Coefficient of determination (R Square) R^2^ = 0.296						
Corrected determination coefficient (Adjusted R Square) R^2^ = 0.257						
Significance of the regression equation F(2. 197) = 9.331 p < 0.0003 Std. error of the estimate: 18.657						
Independent (predictor) variables	BETA (β)	Std. Err. of Beta(β)	Std. B (β)	Std. Err. of B (β)	t (132)	p-level
Sc (Schizophrenia)	0.412	0.161	0.752	0.302	2.515	0.01
Pa (Paranoia)	0.160	0.160	0.310	0.314	1.010	ns.

Dependent (criterion) variable: indirect self-destructiveness (CS-DS)						
Coefficient of multiple regression R = 0.506						
Coefficient of determination (R Square) R^2^ = 0.257						
Corrected determination coefficient (Adjusted R Square) R^2^ = 0.219						
Significance of the regression equation F(2. 197) = 6.317 p < 0.004 Std. error of the estimate: 19.001						
Independent (predictor) variables	BETA (β)	Std. Err. of Beta (β)	Std. B (β)	Std. Err. of B (β)	t (132)	p-level
Pa1 (Persecutory ideas)	0.335	0.161	0.475	0.210	2.128	0.04
Pa3 (Naiveté)	−0.260	0.150	−0.440	0.261	−1.690	ns.

Dependent (criterion) variable: indirect self-destructiveness (CS-DS)						
Coefficient of multiple regression R = 0.633						
Coefficient of determination (R Square) R^2^ = 0.398						
Corrected determination coefficient (Adjusted R Square) R^2^ = 0.382						
Significance of the regression equation F(1. 197) = 24.840 p < 0.00001 Std. error of the estimate: 16.891						
Independent (predictor) variables	BETA (β)	Std. Err. of Beta (β)	Std. B (β)	Std. Err. of B (β)	t (133)	p-level
Sc2B (Lack of ego mastery. conative)	0.632	0.120	1.220	0.240	4.980	0.00001

## Discussion

It is difficult to refer to results of other research because it has not been carried out. The only studies that were conducted focused solely on isolated behaviours, which are now called indirectly self-destructive, rather than on indirect self-destructiveness as a generalized tendency in patients with schizophrenia.

Received results carry important implications for the nature of indirect self-destructiveness in patients with schizophrenia.

As mentioned earlier, a vast majority of research on indirect self-destructiveness concerned mainly the population of mentally healthy individuals. In an attempt to answer the question whether indirect self-destructiveness, as a generalized behavioural tendency, can be reduced to a specific syndrome in the nosological classification, Suchańska examined relationships between indirect self-destructiveness and MMPI scales. In a population of healthy subjects, she noted significant correlations between indirect self-destructiveness and certain clinical scales: Ma (hypomania; 0.533), PD (psychopathic deviate; 0.531), Sc (schizophrenia; 0.501) [3]. As can be seen, the associations between clinical scales and indirect self-destructiveness in the healthy population were distributed differently than in the case of patients with schizophrenia. Namely, in studies on healthy subjects, the highest coefficients were found between indirect self-destructiveness and the scales of Ma and PD. Those findings may reflect a relationship between the pleasure principle (which individuals with psychopathic disorders are remarkably guided by) and immediate gratification, and indirect self-destructiveness.

However, our results showed that patients with Sc displayed the highest correlation coefficients between indirect self-destructiveness and psychotic scales, i.e. Sc and Pa. Therefore, the results indicated associations between indirect self-destructiveness and symptoms of schizophrenic and paranoid disorders (for which the subjects were treated). Significant correlations were also found between indirect self-destructiveness and all the subscales of the Pa and Sc scales.

In order to identify factors that determined indirect self-destructiveness in schizophrenic patients, the stepwise multiple regression analysis was performed, in which independent variables were the MMPI clinical scales and subscales.

As shown in Table 3, only two of the clinical scales remained in the regression equation: the scale of Sc and Pa, of which Sc proved significant for indirect self-destructiveness. Thus, among the studied disorders, schizophrenic disorders best explain the indirect self-destructiveness syndrome in patients with schizophrenia.

The diversity, richness and multi-faceted nature of schizophrenic symptoms were also reflected in the structure of MMPI-2, which comprises several subscales of the Sc and Pa clinical scales. Along with the identified associations between indirect self-destructiveness and Pa and Sc, it seemed worth trying to determine which symptoms and disorders of the rich symptomatology of Sc were significant predictors of the indirect self-destructiveness syndrome in patients with schizophrenia. Table 2 shows that indirect self-destructiveness significantly correlated with all the subscales of those clinical scales; all coefficients had plus signs, except for subscale Pa3 (Naiveté) which had the minus sign. The content analysis of those subscales allows to obtain knowledge of many aspects of the indirect self-destructiveness tendency in patients with schizophrenia. Pa1 include, among others, a feeling of being harmed by life, which may cause a suspicious attitude towards people (Pa3-Naiveté, negative coefficient); in turn, the item of the CS-DS which received the highest score in both males and females with Sc reads as follows: “It’s easy to be harmed by life”. Thus, it can be assumed that schizophrenic patients’ sense of injustice was very important for the shaping of their indirectly self-destructive tendencies. Of similar significance was the correlation between indirect self-destructiveness and Sc scale subscales: a sense of injustice (of life) and being misunderstood by others (Sc1A-social alienation), feeling of the lack of meaning of life (Sc1B-emotional alienation), sense of unreality of what is happening around (Sc2A-lack of ego mastery, cognitive), feeling of lack of control over one’s own emotions and impulses (Sc2C-lack of ego mastery, defective Inhibition), and bizarre sensory experiences (Sc3). What deserves special attention is the subscale which showed the strongest relationship, i.e. Sc2B (lack of ego mastery, conative) and included the perception of life as a tremendous effort, lack of satisfaction with own actions, and lack of hope for improvement.

Apart from the presented correlations, shares of the individual variables, i.e. subscales of the Pa and Sc clinical scales, in the shaping of the indirectly self-destructive tendency in patients with Sc should be assessed. Table 3 shows that, in the case of the Pa-scale subscales, two variables (Pa1 and Pa3) remained in the regression equation, of which an important factor in determining indirect self-destructiveness proved to be Pa1, whose main component is the feeling of being harmed by life. The implication of the share of another variable which remained in the regression equation of the clinical scale subscales, i.e. lack of ego mastery, conative (Sc2B), the core of which is experiencing life as a tremendous effort, is similar. It should be borne in mind that out of all the diagnostic items of indirect self-destructiveness, patients with Sc (regardless of sex) rated the most highly the statement that it is very easy to be harmed by life. Patients with Sc hold a strong conviction that they have been harmed by life.

Those indirect self-destructive tendencies connected with psychotic disorders/symptoms may inhibit and delay recovery.

## Conclusions

Schizophrenic disorders are a predictor explaining the indirect self-destructiveness syndrome in those patients. Among schizophrenic and paranoid disorders and symptoms, an important factor in determining indirect self-destructiveness proved to be Pa1, especially the sense of injustice and experiencing life as an enormous effort.

Therapeutic work should take into account also that aspect of psycho(patho)logical functioning, i.e. indirect self-destructiveness, which is strongly associated with schizophrenic and paranoid disorder/symptoms.

After all, it is known that indirect/chronic self-destructiveness may evolve into direct/acute self-destructiveness.

## Abbreviations

CS-DS: Chronic Self-Destructiveness Scale
MMPI: Minnesota multiphasic personality inventory
Hd: Hypochondriasis
D: Depression
Hy: Hysteria
PD: Psychopathic deviate
MF: Masculinity–femininity
Pa: Paranoia
Pt: Psychasthenia
Sc: Schizophrenia
Ma: Hypomania
SI: Social introversion
Pa1: Persecutory ideas
Pa2: Poignancy
Pa3: Naiveté
Sc1A: Social alienation
Sc1B: Emotional alienation
Sc2A: Lack of ego mastery, cognitive
Sc2B: Lack of ego mastery, conative
Sc2C: Lack of ego mastery, defective inhibition
Sc3: Bizarre sensory experiences
R: Coefficient of multiple regression
R^2^: Coefficient of determination (R square)
β: Beta
